# Shell Wounds in the Bombardment of San Juan De Porto Rico

**Published:** 1898-09

**Authors:** 


### Shell Wounds in the Bombardment of San Juan De Porto Rico.

The casualties in the bombardment of San Juan amounted to one man killed and five wounded. The action lasted nearly three hours, the following vessels participating: The cruisers New York, Detroit and Montgomery; the battleships Iowa and Indiana; the monitors Terror and Amphitrite, and the torpedo boat Porter. Only two shells from the enemy's batteries struck the ships, one shell striking the New York, and one the Iowa. The shell which injured the New York was a cast-iron eight-inch one. It struch a stanchion and burst, the fragments flying in every direction. A correspondent writes to the *Medical Record* that one fragment struck a man just behind one ear and emerged at the other side of his head. He died almost instantly. Another fragment struck another man in the lower third of the thigh. The missile entered posteriorly and emerged anteriorly. The femur was comminuted. The fragments were resected and the bone ends wired together by the surgeons on board the New York. The same man received a flesh wound in the calf of the other leg from another fragment of the same shell. The third man wounded by this shell received a flesh wound only. The shell which struck the Iowa entered the superstructure and burst. A piece of this shell wounded a marine in the right elbow from behind. The external condyle of the humerus, the head of the radius, and part of the ulna were shattered and carried away. Efforts are being made to save the arm. Another man was wounded by a splinter from one of the steel girders of the superstructure, which was detached by the shell. He received a lacerated and contused wound of the back, which laid bare the ribs, but did not injure them or the deeper structures. Another man was wounded in the foot, but with no bone involvement. All the wounds are doing well and are aseptic seven days after the receipt of the injuries.—*Gaillard Medical Journal.*

